# Increase in dental caries and change in the socioeconomic profile of families in a child cohort of the primary health care in Northeast Brazil

**DOI:** 10.1186/s12903-019-0871-9

**Published:** 2019-08-14

**Authors:** Márcia Maria Dantas Cabral de Melo, Wayner Vieira de Souza, Paulo Sávio Angeiras de Goes

**Affiliations:** 1 Department of Clinical and Preventive Dentistry, Federal University of Pernambuco, Av. Professor Moraes Rego, s/n. Cidade Universitária, Recife, PE CEP: 50,670-420 Brazil; 2 Aggeu Magalhães Institute, Oswaldo Cruz Foundation, Av. Moraes Rego, s/n. Cidade Universitária, Recife, PE CEP: 50670-420 Brazil; 3 Department of Clinical and Preventive Dentistry, Federal University of Pernambuco|, Av. Professor Moraes Rego, s/n. Cidade Universitária, Recife, PE CEP: 50,670-420 Brazil

**Keywords:** Dental caries, Incidence, Children, Social conditions, Primary health care

## Abstract

**Background:**

Factors associated with increases in dental caries and changes in the family socioeconomic profile were investigated in a paediatric primary health care (PHC) cohort in Northeast Brazil during the implementation of social and income transfer programmes.

**Method:**

A prospective analytical study compared data from two surveys on caries in primary dentition conducted in 2006 (age: 18–36 months, *n* = 1045) and 2010 (age: 5–7 years). Data from the sample recruited and re-examined in 2010 (*n* = 469) were analysed. Prevalences (P) and the mean primary decayed, missing and filled teeth (dmft) index, cumulative incidence and mean increase were calculated. Differences (*p* ≤ 0.05 and 95% CI) in dmft ≥1 were identified via McNemar’s test. Differences in the mean dmft were evaluated according to socioeconomic variables (Kruskal-Wallis test and *p* ≤ 0.05). Multivariate analysis with a negative binomial model was used for the risk factors associated with increasing dmft. In the univariate analyses, nonparametric methods (Kruskal-Wallis test) were used to compare subsamples. Variables with *p* ≤ 0.20 were included in the multivariate model and retained when *p* ≤ 0.05.

**Results:**

The prevalence and mean dmft (18–36 months and 5–7 years: *p* = 28.6 and 68.9%, mean = 1.01 and 3.46, respectively) and variation in mean dmft changed significantly (*p* < 0.005) with the education level and occupation of the mother; the prevalence and mean dmft were lower for higher maternal education level and maternal participation in the labour market. The cumulative incidence and mean increase in dmft were 8.71% and 2.45, respectively. Common risk predictors for increases in caries were consumption of sweets (RR = 1.53, 95% CI 1.09–2.14) and attendance at public schools (RR = 1.49, 95% CI: 1.81–1.89). Use of private clinical services was a protective factor (RR = 0.68, 95% CI 0.54–0.87).

**Conclusion:**

Increases in caries were observed despite positive changes in the distribution of socioeconomic indicators for the analysed children’s families. The risk factors identified for the increase in caries suggest ongoing problems regarding the effectiveness of intersectoral and health measures for controlling caries in populations exposed to PHC programmes.

## Background

Dental caries attack in childhood is a frequent cause of pain, discomfort and feeding difficulties that compromise quality of life and the biopsychosocial development of children.

Despite improvements in the oral health of children with primary dentition, the highest rates of dental caries persist among socioeconomically disadvantaged children [1, 2]. These facts suggest that health inequities are an expression of the dynamics of the social, political, economic and cultural processes of each society [3]. As well as the increasing socioeconomic inequality observed among social classes, regions of the world and within countries [3].

In Brazil, the epidemiological profile of caries in children with primary dentition is characterized by a high prevalence of untreated caries, early onset, increased severity with increasing age and a heterogeneous distribution pattern of caries between regions of the country [4]. National data from 2010 indicate that approximately 50% of children under 5 years of age require treatment, and the “caries” component accounted for 84.5% of the primary decayed, missing and filled teeth (dmft) index. The mean dmft ranged from 2.80 in 2003 to 2.43 in 2010, representing a reduction of only 13.2% in 7 years, whereas at 12 years of age, the reduction in the DMFT was 26.2% [5].

In this context, the Brazilian Health System has been pursuing the implementation of policies governed by the principles of universality and comprehensiveness, with a focus on primary health care (PHC). In that line, new oral health guidelines have been adopted to reverse the unfavourable oral health conditions to which significant population groups are subjected, including children [4, 6].

In that context, in 2006, a cohort of preschool children, aged between 18 and 36 months, who were seen at PHC units of an important metropolitan centre in Northeast Brazil, was used to investigate the risk factors associated with the prevalence of dental caries in the primary dentition. The findings aimed to help support local health policies [7].

Over time, this problem has been investigated using cross-sectional studies. Although such studies provide an overview of reality, they cannot elucidate causal paths. This sometimes obscures or provides evidence for factors that are stronger in the analyses when time is not considered a relevant factor, such as local factors.

This study aimed to present the risk factors related to the increase in dental caries in the primary dentition identified between 2006 and 2010 in this paediatric cohort; additionally, the role of changes in the family socioeconomic profile during a period where social and income transfer programmes were implemented in the country was evaluated.

## Methods

The present study is defined as a prospective analytical study that was conducted in 2006 and 2010 in a cohort of children seen at PHC services [7].

This study was developed in family health units (FHUs) of the PHC system in two regions of Recife, Pernambuco State capital, located in Northeast Brazil, which corresponds to Health Districts (HDs) II and IV. In 2010, the city contained 1,537,704 inhabitants [8]. The city is completely urban and is heterogeneous in terms of the housing, garbage disposal and sewage conditions, which show unequal patterns in the living and spatial occupation conditions of the city’s inhabitants [9]. In addition, the public water supply system is irregular and do not have fluoridation. Despite improvements in social indicators, the Gini coefficient for household income per capita, which measures income concentration, increased from 0.6739 in 1991 to 0.6789 in 2000 and to 0.6894 in 2010, indicating an increase in income concentration of 2.3% in the city [9]. The HD areas studied are composed of communities with the greatest needs in terms of housing conditions and essential public services, where more than 20% of the residents live on less than the minimum wage (R$ 350.00 in May 2006 and R$ 510.00 in 2010, [10].

The study population consisted of the total sample of children from the 2006 cross-sectional study [7], which was conducted when they were between 18 and 36 months old, and who were recruited to be re-examined in 2010 when they were aged between 5 and 7 years. The sample size calculation of the 2006 survey was based on a 27% caries prevalence at 18 and 36 months, according to 2003 national data [11] for Northeast Brazil. Assuming a sample error of 5%, a confidence interval of 95% and a design effect equal to 1, a sample size of 1.800 children was estimated for both HDs. The sample was allocated equally to each HD (*n* = 900 in each district). Then, in each HD, 9480 families were selected proportionally to the number of families enrolled in the FHUs of each HD. Next, all children who met the age criterion of the study were selected from these families. A total of 1415 individuals from both HDs between 18 and 36 months of age participated in the survey, representing an overall participation rate of 78.6% [7].

This study included the records of the children who were recruited for the 2006 study and re-examined in 2010 and whose data were stored and validated for analyses in the databases of those surveys. Considering the inclusion criteria, the analysed sample corresponded to a total of 469 children [224 individuals (47.8%) from HD II and 245 (52.2%) from HD IV]. Regarding the age distribution, in 2010, there was a predominance of children older than 7 years (*n* = 259), which corresponded to 55.2% of the sample.

The data obtained through clinical exams were used to measure the dmft index and the structured interviews (questionnaires) applied in 2006 and 2010. The data quality control was performed by analysing the reproducibility of the observations made by the examiners and by the review and pre-test of the questionnaire. In both surveys, the same guidelines were followed for validation of the questionnaire and for the diagnosis of caries [7], which were based on international guidelines [12]. For the exams, the children were accompanied by their guardians, who authorized the exams. Children with identified dental needs were scheduled for treatment. In the 2006 study, 31 examiners and 31 auxiliaries belonging to the FHUs of the two HDs participated in the data collection. In 2010, data were collected by 34 examiners and 34 auxiliaries from the same FHUs as in the 2006 study. They received 18 h of training and calibration, the results of which were assessed using the overall percent agreement (OPA) and the Kappa coefficient. The overall values obtained reached recommended reliability scores for epidemiological surveys of caries [12]. In both surveys, the lowest and highest OPA and Kappa values observed (95% CI) for inter-examiners were 92.1% and 0.78 (95% CI: 0.71–0.75) and 97.0% and 0.81 (95% CI 0.78–0.83) in 2006 and 89.7% and 0.75 (95% CI 0.72–0.78) and 95.9% and 0.90 (95% CI, 0.76 to 0.81) in 2010, respectively.

To analyse the risk factors related to caries incidence, a risk model was constructed based on the increase (counting) in the dmft between 2006 and 2010. The independent variables used to explain the outcome were selected and recategorized from the original questionnaires and were socioeconomic factors (sex, age, mother’s education level, mother’s occupation, father’s occupation, number of people in the household, length of time the child lived in the area), oral care factors (consumption of sweets between meals, frequency of brushing) and use of education and health services (type of daycare/school, use of dental services of the PHC units, use of other dental services) available in both study rounds.

A data analysis was first performed to identify selection biases and to prove the validity of the sample data. A comparative analysis was performed between the profile of the 2006 study population that was recruited (*n* = 469) to be re-examined in 2010 and the portion of the sample that was not recruited (*n* = 946)…

For the dmft index analyses, the prevalences (p) and means for the periods of 2006 and 2010 were calculated. The prevalences (dmft ≥1) were calculated by generating their point estimates together with their 95% confidence intervals (95% CIs). McNemar’s test at a significance level of 5 and 95% CI was used to test for significant differences between the prevalences observed in the two periods. We also estimated the annual reduction in the percentage of caries-free individuals (dmft = 0), calculating the mean annual number of new cases (dmft ≥1) divided by the number of caries-free cases in 2006 (nf). The mean annual number of new cases was calculated by the difference between the number of positive cases (dmft ≥1) in 2006 (n_1_) and 2010 (n_2_). The time interval (t) between the two surveys was approximately 4.5 years. Two periods of data collection lasted from June 2006 [beginning of the first data collection] to December 2010 [end of second data collection]). The annual reduction in the percentage of caries-free individuals (FCR%) was estimated using the following expression:

$$ FCR\left(\%\right)=\frac{\left({n}_2-{n}_1\right)\div t}{nf} $$

The means and standard deviations of the dmft were estimated, and the mean differences were evaluated according to the categories of socioeconomic variables related to the mother’s education level, parents’ occupation and number of people in the household, according to the ages of the children and the year of study. To identify the statistically significant differences in the distribution of dmft between the groups, the Kruskal-Wallis test was used with a significance level of 5%.

To identify the risk factors associated with the increase in dmft (response variable), a multivariate analysis using a negative binomial model was used. As a first step, univariate analyses were performed to test the association between the independent variables and the increase in dmft, using non-parametric methods (Kruskal-Wallis test) to compare several subsamples and because of the non-normality of the analysed distributions. The variables that showed a significant association with *p* ≤ 0.20 were included in a multivariate negative binomial regression model. The final model was established through the stepwise removal of the variables with *p* ≥ 0.05.

A negative binomial model was used to analyse the increase in dmft because this type of model is recommended for cases where the dependent variable is a count of incident episodes in which “*overdispersion*” is observed in its distribution. In addition, the knowledge already acquired about the probability distribution of this dmft index reinforces this choice since the negative binomial distribution produces a compatible result with the literature on the distribution characteristic of this disorder in the population [13].

Notably, this model used a robust variance estimator and did not consider zero inflation, since all null increases are true as they result from clinical exams.

The statistical software used in the analyses was STATA version 12; Copyright 1985–2011; StataCorp; 4905 Lakeway Drive; College Station, Texas 77,845 USA; 800-STATA-PC; http://www.stata.com; stata@stata.com; 979–696-4600; 979–696-4601 (fax).

## Results

The comparative analysis of the data do not show significant differences between from the two samples (recruited *n* = 469 and non-recruited *n* = 946).

The analysis of the dmft index showed a mean dmft of 1.10 for the 18- and 36-month-olds, in which 29.6% (95% CI: 25.4–33.8%) of the sample had a dmft ≥1. Between 5 and 7 years of age, there was a reduction in the number of children with a dmft = 0, and the percentage of children with a dmft ≥1 was 68.9% (95% CI 64.7–73.1%), with a mean dmft of 3.46 teeth with caries. The mean annual incidence rate or mean cumulative incidence (4.5 years) was 12.4%, reflecting the significant difference in prevalence between the two periods (Table 1).

**Table 1:** Increase of dental caries prevalence and percentage reduction of caries-free individuals between 2006 and 2010

Total n	Year 2006 Age: 18–36 months			Year 2010 Age: 5–7 years			Differences		*P*-value^c^	FCR(%)
	dmft^b^ = 0	dmft ≥ 1	p_1_ a% (95% CI)	dmft = 0	dmft ≥ 1	p_2_ a% (95% CI)	n_2_- n_1_	(p_2_- p_1_)% (95% CI)		
469	330	139	29.6% (25.4–33.8)	146	323	68.9% (64.7–73.1)	184	39.2% (34.8–43.6)	*p* < 0.001^c^	12.4% per year

^a^p_1_, p_2_,: caries prevalence

^b^Caries index, dmft (decayed, missing and filled teeth) index

^c^The significance (*p* ≤ 0.05) of the dmft scores ((p_2_- p_1_) as measured by McNemar’s test

**Fig. 1:**
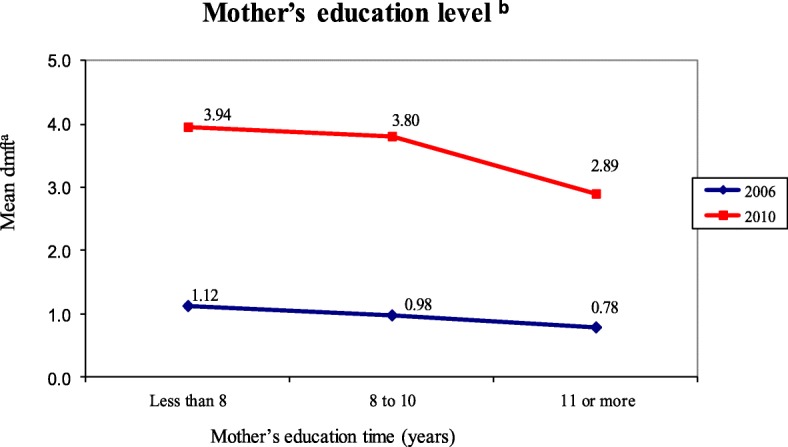
Variation in mean dmft according to the categories of the mother’s education level. ^a^Caries index, dmft (decayed, missing and filled teeth) index. ^b^The statistical significance (*p* ≤ 0.05) of the dmft distribution was assessed using the Kruskal-Wallis test

**Fig. 2:**
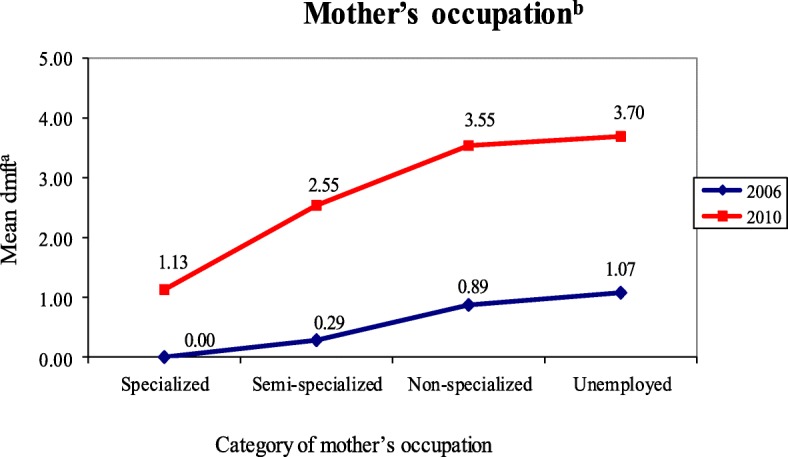
Variation in mean dmft according to the categories of mother’s occupation. ^a^Caries index, dmft (decayed, missing and filled teeth) index. ^b^The statistical significance (*p* ≤ 0.05) of the dmft distribution was assessed using the Kruskal-Wallis test

Figures 1 and 2 present the results of the analysis of the variation in mean dmft according to the categories of variables related to the mother’s education level and occupation for the ages and study year. These two variables were those that presented significant changes throughout the study period.

In 2006, no statistically significant difference was observed between the mean dmft estimated for each of the categories of mother’s education level (*p* = 0.201). However, the highest mean dmft values (1.12 ± 2.19) were observed for less than 8 years of education, which had a higher percentage of mothers (51.2%) in this precarious condition. The lowest mean values of dmft (0.78 ± 2.02) were observed in children whose mothers had the highest education levels (24.0%). In 2010, a statistically significant difference was observed (*p* = 0.002). The highest dmft mean (3.94 ± 3.02) belonged to children whose mothers had only a few years of education (< 8 years). However, this category had the lowest number of mothers (10.1%). The results showed that 41.1% of the mothers with low levels of education, as registered in 2006, migrated from less than 8 years of education to the categories of 8 to 10 years (52%) or 11 years or more of education (37%) in 2010.

The variation in the dmft means observed in 2006 for each of the four categories of mother’s occupation showed no significant differences (*p* = 0.272). Most mothers (75.8%) did not have a job, and their children, as assessed in the first survey, had the highest mean dmft (1.07 ± 2.19). Zero values for dmft were observed in children whose mothers had specialized jobs. In 2010, there was a positive (upward) change in the percentage distribution of this variable referring to the inclusion of the mothers of the studied children in the labour market. This change was followed by a decrease in caries severity (dmft) observed according to the occupation category of these mothers [unemployed (47.1%): dmft = 3.70 ± 3.93; non-specialized (38.6%): dmft = 3.55 ± 3.31; semi-specialized (12.6%): dmft = 2.55 ± 3.17; specialized (1.7%): 1.13 ± 2.10]. The differences were significant (*p* = 0.031).

Table 2 shows the results of the univariate analyses for the increase in dmft. For the total sample, the mean increase in dmft was 2.45 with a standard deviation ±2.56. This mean increase is due to the occurrence of 1150 new cases (teeth) in 469 children, with implies in an annual incidence rate of 0.54 tooth per child per year. Of the 12 variables selected, seven were included in the subsequent multivariate analysis since they presented a *p* value ≤0.20 (sex, mother’s education level, mother’s occupation, consumption of sweets between meals, frequency of brushing, type of school and private dental service). Among these variables, “mother’s education level” (*p* = 0.0002), “consumption of sweets” (*p* = 0.0001) and “type of school” (*p* = 0.0001) showed the strongest association with the increase in dmft. “Sex” also presented a *p* value ≤0.05.

**Table 2:** Univariate analysis between the increase in dental caries and risk factors of the sample

Variable	Sample (n)	Increase in dmft^a^ (Mean ± SD)	*P*-value^b^
Total sample	469	2.45 ± 2.56	
Sex			
Female	227	2.70 ± 2.55	0.0138^b^
Male	242	2.42 ± 2.56	
Total	469	2.45 ± 2.56	
Age			
5 years	45	2.82 ± 2.93	0.6954
6 years	165	2.45 ± 2.71	
7 years	259	2.39 ± 2.40	
Total	469	2.45 ± 2.56	
Mother’s education level			
< 8 years	47	3.00 ± 2.52	0.0002^b^
8 to 10 years	243	2.79 ± 2.68	
11 years or more	175	1.85 ± 2.30	
Total	465	2.46 ± .2.57	
Mother’s occupation			
Specialized	8	1.13 ± 2.10	0.1876^b^
Semi-specialized	58	1.98 ± 2.42	
Non-specialized	178	2.61 ± 2.67	
Unemployed	217	2.49 ± 2.52	
Total	461	2.45 ± 2.57	
Father’s occupation			
Specialized	15	1.80 ± 2.34	0.2891
Semi-specialized	222	2.26 ± 2.46	
Non-specialized	198	2.73 ± 2.71	
Unemployed	28	2.32 ± 2.50	
Total	463	2.45 ± 2.57	
Number of people in the household			
Up to 3	119	2.58 ± 2.80	0.4734
4 to 5	219	2.31 ± 2.51	
6 or more	128	2.55 ± 2.40	
Total	466	2.44 ± 2.56	
Length of time the child lived in the area			0.5902
Less than 5 years	18	2.61 ± 2.52	
5 years or more	451	2.45 ± 2.57	
Total	469	2.45 ± 2.56	
Consumption of sweets between meals			
Never	83	1.73 ± 2.37	0.0001^b^
Sometimes	201	2.16 ± 2.47	
Daily	182	3.11 ± 2.67	
Total	466	2.46 ± 2.57	
Frequency of brushing			0.0294^b^
Never/Sometimes	56	3.13 ± 2.73	
Daily	411	2.37 ± 2.53	
Total	467	2.46 ± 2.57	
Type of school			0.0001^b^
Private	195	1.78 ± 2.31	
Public	260	2.97 ± 2.63	
Total	463	2.45 ± 2.56	
Use of PHC dental services			0.6832
Yes	362	2.48 ± 2.55	
No	107	2.36 ± 2.60	
Total	469	2.45 ± 2.56	
Use of private dental services			0.1265^b^
Yes	82	2.94 ± 2.80	
No	381	2.36 ± 2.51	
Total	463	2.46 ± 2.57	

^a^Caries index, mean dmft (decayed, missing and filled teeth) index, SD standard deviation

^b^The significance (*p* ≤ 0.05) of the increment dmft scores as measured by the Kruskal-Wallis test

The final model of the multivariate analysis concerning the independent risk factors for the increase in caries in the total sample is presented in Table 3. The test for assessing the goodness-of-fit of the negative binomial model compared to the Poisson model confirmed the adequacy of the model used (α = 0.89, 95% CI 0.70–1.13, *p* ≤ 0.05).

**Table 3:** Final model of risk factors related to the increase in dental caries

Variables/Categories	^a^RR^crude^ RR and 95% CI	*P*-value^c^	^b^RR^adjusted^ RR and 95% CI	*P*-value^d^
Sex				
Female	1	0.096	1	0.099
Male	1.18 [1.01–1.43]		1.18 [0.97–1.43]	
Mother’s education level				
< 8 years	1.42 [0.98–2.07]	0.063	1.55 [1.05–2.01]	0.010^d^
8 to 10 years	1.25 [0.96–1.62]	0.100	1.28 [1.00–1.65]	0.052^d^
11 years or more	1.00	–	1	
Mother’s occupation				
Specialized	1		#	#
Semi-specialized	1.68 [0.52–5.41]	0.381		
Non-specialized	1.85 [0.59–5.78]	0.289		
Unemployed	1.83 [0.59–5.77]	0.297		
Consumption of sweets between meals				
Never	1		1	
Sometimes	1.17 [0.82–1.67]	0.400	1.12 [0.79–1.58]	0.527
Daily	1.60 [1.12–2.27]	0.009	1.53 [1.09–2.14]	0.014^d^
Frequency of brushing				
Never/Sometimes	1.13 [0.86–1.48]	0.376	#	#
Daily	1			
Type of school				
Public	1.51 [1.19–1.92]	0.001	1.49 [1.18–1.89]	0.013^d^
Private	1		1	
Use of private dental services				
Yes	0.69 [0.54–0.89]	0.004	0.68 [0.54–0.87]	0.002^d^
No	1			

^a^*RR*^*crude*^ crude relative risk

^b^*RR*^*adjusted*^ adjusted relative risk

^c,d^The statistical significance (*p* ≤ 0.05) of the multivariate negative binomial regression model

The variables related to the occupation of the mother and the control of bacterial plaque were removed from the final model. The variable “sex” remained in this final model at the established limit (*p* = 0.099). Only a proxy variable of living conditions from the socioeconomic block, “mother’s education level”, was an explanatory factor for the increase in caries in this population. Children whose mothers had less than 8 years of education had a higher risk of developing caries (RR = 1.55 and 95% CI 1.05–2.01) compared to children of mothers with higher education levels. The consumption of sweets between meals was a risk factor for an increase in caries, and children with this daily habit had a higher risk of developing caries (RR = 1.53 and 95% 1.09–2.14) than did children without this habit.

The two variables related to the type and use of education and health services were associated with an increase in caries. Children attending public schools (RR = 1.49 and 95% CI 1.81–1.89) had a higher risk of caries than those attending private schools. However, children who were treated at private dental services, in addition to those treated in PHC, had a negative association with the outcome, i.e., it was a protective factor (RR = 0.68, 95% CI 0.54–0.87).

## Discussion

This study ratifies the role of socioenvironmental determinants in the increase in caries in children treated in PHC units in Northeast Brazil and sheds light on the limitations of traditional oral health education approaches and dental care provided by the Family Health Strategy.

The dmft analyses of the total sample showed incidences of caries that were higher than the figures from the 2003 [11] and 2010 [5] national surveys. In 2006, 70.4% of children between 18 and 36 months old were free of caries. Of these children, only 44% remained free of caries at 5 to 7 years of age, when they presented a higher mean dmft than the value recorded by the 2010 national survey of 5-year-old children living in the poorest regions of Brazil [5]. Notably, the study sample included only socially vulnerable families, whereas the national survey included individuals from different social strata, which may result in lower dmft values. Furthermore, the values agreed with those obtained in other studies with similar populations [14, 15].

Although health policies implemented in Recife are in line with national guidelines to address health problems widely, for children aged 5 to 7 years old, the percentage values ​​of the caries component predominated over the others, similar to that observed when they were 18 to 36 months and in other similar studies [11, 14, 15]

The increase in caries observed suggests problems with the effectiveness of the health measures adopted to control the disease and demonstrated the role of socio-environmental determinants even in a population that is exposed to PHC programmes. Other national studies have shown positive associations between the development of new early caries and conditions related to socioeconomic disadvantages, the housing environment, access to health and education services and programmes and behavioural aspects [16–19]. These associations are present in countries of different levels of development [1, 13, 15, 20]. This may point to agglomeration with the consequent synergism of risk factors in areas of social vulnerability [21].

However, changes were observed in the distribution of most family socioeconomic variables of the children, suggesting some improvement in living conditions. An example of how these improvements can be expressed in terms of oral health of the sample is the finding that children whose mothers were in better categories in terms of education levels and occupation in 2010 had a lower mean dmft compared to children of mothers who remained in the lower strata of these two variables. These data corroborate studies that address the relationship between the socioeconomic position occupied by the individuals and the different gradients of caries among the populations [1, 3, 4] and agree with studies that show that individuals who had childhood caries and whose families improved socially reached better levels of oral health at older ages [22].

In addition, these findings should be analysed in light of the social transformations that have occurred in Brazil since 2003, mainly the implementation of redistributive policies [8], which have had positive effects on socioeconomic, health and oral health indicators [6, 8, 23]. Between 2000 and 2011, an analysis of changes in the labour market in the country showed that there was a reversal in the unemployment and informal employment trend, which, although not homogeneous, improved particularly in the Northeast [23]. In Recife, between 2000 and 2010, the percentage of the economically active population remained essentially the same. Simultaneously, their unemployment rate was reduced by 8.6%, but the income concentration increased by 2.3%. There was also an increase in the mean number of years of education in the adolescent and adult population, but with regional asymmetries [8]. For Recife, data from 2010 reported that more than 50% of the population aged 18 or over had completed elementary or secondary school, in addition to an increase in the proportion of children aged 5 to 6 who were enrolled in school [8, 9].

The multivariate analysis, which identified factors associated with the increase in caries, demonstrated the persistence of factors both at the onset of the disease and throughout its development. Contrary to the baseline of this study, when the sample population was between 18 and 36 months old and when there was an association between dmft and the older age group [7], age was not associated with this problem in the present study. No stratum of this variable was statistically significant, despite the observed increase in mean levels of caries with increasing age. The passage of time has been associated with worsening oral health conditions, which ultimately represents an accumulation of risks from different domains [3, 19, 21] and is consistent with the levels of dmft recorded between 2006 and 2010.

The mother’s education level, which is a proxy for living conditions, was a statistically significant predictor for the increase in caries, in accordance with other prospective national [16, 17, 24, 25] and international [20, 26, 27] studies. Conversely, when the sample population was 18 to 36 months old, the mother’s education level was not associated with caries, which seems to reinforce discussions about the accumulation of risks over time for the onset of the disease [21].

Another variable used as a proxy for socioeconomic conditions, school type, was a risk factor for the increase in caries, similar to that observed in 2006 [7]. The children enrolled in public schools had an RR of developing caries of approximately 50% during the study period compared to the children who attended private schools. The effect of housing environment factors, coupled with the possible geographical location of the school, may influence this prediction. These results agree with other national studies that showed a higher probability of caries in children enrolled in public schools compared to those enrolled in private schools [14, 28]. However, a study in southern Brazil found that not attending public preschools was a more proximal factor in the development of caries at 6 years of age and was mediated by factors related to low maternal education and family income [29].

These results also reveal that the recent proposals of intersectoral policies for the school environment [30] do not favour reducing this risk factor, which has remained associated with a dmft ≥1 and an increase in caries in the children followed up between 2006 and 2010. This suggests that the public education policies for the areas studied are not effectively incorporating actions that promote oral health, which should be valued in view of the effect of caries’ severity on school performance [7, 31]. A study in Recife demonstrated that children with severe caries may have their learning compromised as a result of missing classes due to toothache and interference with their quality of life [32, 33]. In a study in Belém do Pará, the adoption of intersectoral promotional measures enabled the stabilization low levels of caries in children from public preschools who were followed up for 4 years [34].

Despite the improvements in access to restorative treatment in this population, with 20% of teeth affected by caries restored in 2010, using the FHU dental services did not constitute a risk factor associated with the increase in caries. Nonetheless, one study reported that between 2001 and 2007, preventive dental procedures outnumbered curative procedures in the FHUs of Recife’s PHC [35]. However, those children who used private dental services were protected from the risk of acquiring new carious lesions.

The strong association found between the increase in caries and the use of dental services in FHUs reveals difficulties for children to access care actions and monitor risk indicators in a timely manner to remain free of caries and/or prevent the development of new lesions, despite the fact that this age group is among those prioritized for Brazilian public dental care [4, 5].

This result also reveals heterogeneity in the socioeconomic conditions of families considered homogeneous, in agreement with studies that show that individuals with higher socioeconomic and educational levels tend to seek health services earlier and make better use of dental services, even in regard to services whose access is more difficult, such as specialized care [36]. In the United States, a lower probability of caries was identified in children with dental insurance than that in uninsured children [37]. The use of dental services by poor children and at earlier ages is an unresolved and discriminating issue that has been pointed out by national studies [33, 38–40] and other similar international studies [1, 26, 31, 41].

Proximal determinants of the health-disease process that affect the oral health of children, both biological and behavioural, have been well described [16, 19, 25, 29]. A more proximal factor of the disease that confers validity to this study was the universal finding of the association between the frequent and daily consumption of sweets between meals and caries in the primary dentition [17, 42]. This finding was observed in the final risk model for the increase in caries when the sample population reached 5 to 7 years old and was also a major risk factor for dmft ≥1 when the sample population was 18 to 36 months old [7]. In light of new studies, it is important to emphasize the difficulties of adopting changes in individual habits that are harmful to oral health. More comprehensive intervention plans that exert influences on the family’s dietary behaviours [7, 31] and that incorporate subjective and cultural aspects involved in the development of eating habits that begin in childhood are necessary [31].

The results of this study should be analysed in light of its limitations. With regard to selection bias, methodological care was taken to ensure the internal validity of the data and to ensure the accuracy of the study since the sample size recruited from the initial 2006 study and re-evaluated in 2010 was smaller. Of the two variables that presented significant differences, only one remained in the risk model for the increase in caries in the sample. Although the significant differences found indicate the existence of possible selection biases, one should consider the influence of the increased power of the tests when working with large samples, as in this study. The heterogeneity of the supposedly homogeneous sample was controlled insofar as socioeconomic condition data were collected.

Despite the adoption of the multivariate analysis with a negative binomial model, which is indicated as appropriate for modelling the increase in caries [43], one should consider the limitations of the analytical method used regarding the possibility of presenting the complexity of the studied process because all explanatory variables were at the individual level, that is, a multilevel analysis was not performed. Another limitation of this study is the possibility of not having detected new cases of caries because of the non-inclusion of the dmfs index, whose unit of measure is the dental surface. This fact would refine the calculation and the analysis of incidence with the negative binomial model adopted.

Attention should be paid when generalizing the results. The studied population is specific and comes from poor urban areas of a Northeast Brazilian city, under the influence of PHC policies, and it is important to compare it with similar populations.

## Conclusion

Increases in caries were recorded despite the positive changes in the distribution of socioeconomic indicators of the families of the children who were followed up. The variables that composed the risk model for the increase in caries suggest the persistence of problems with the effectiveness of intersectoral and health measures to control the disease in a population exposed to PHC programmes. In addition, it is paradoxical that the use of private services, commonly of restricted biomedical approach, has constituted a protective factor for the increase in caries in these children, who should have full use of the actions offered by the PHC in the study areas.

## Data Availability

The datasets used and/or analysed during the current study are available from the corresponding author on reasonable request.

## Abbreviations

95% CI: 95% confidence interval
dmft: decayed, missing and filled teeth index for primary dentition
FHU: Family health units
HD: Health district
OPA: Overall percent agreement
P: Caries prevalence (dmft ≥1)
PHC: Primary health care
RR: Estimate of relative risks
SD: Standard deviation
